# On Tracheotomy in Epilepsy Laryngea

**Published:** 1854-12

**Authors:** Marshall Hall


### SELECTIONS.

*On Tracheotomy in Epilepsia Laryngea.* By MARSHALL HALL, M. D., F.R.S., &c.

I now resume the question of the institution of tracheotomy in epilepsy. In doing this, I present to the readers of *The Lancet* the results of several years of careful investigation.

I can conscientiously affirm that my hopes of relief in *this direst* of human maladies, from tracheotomy, are more sanguine than ever. I do not, and never did, expect to cure epilepsy, especially epilepsy in its inveterate stages, or with organic lesions, by tracheotomy. I did not expect much from the use of this measure amongst the "incurables" of the lunatic asylum or the workhouse. I did not expect any good results, except by accident, in the cases in which the patient was subjected to tracheotomy without a just and previous diagnosis; for it is still necessary to repeat, that it is for laryngismus, spasmodic or paralytic, and its effects, and not for epilepsy, any more than for apoplexy, that I have proposed tracheotomy as a remedy.

In a word, I proposed tracheotomy for epilepsy in its direst form, short of inveteracy and organic lesion,—in effect, whilst hope still remained. I proposed it for that form—that of the epilepsia laryngea,—assuredly expecting it to reduce it to some other milder abortive form; and my hopes have not been disappointed. In one case,—a case in a lunatic asylum, too—the fits were reduced, by "a moderate and impartial estimate, fifty per cent. in frequency, and seventy-five per cent. in severity;" and were "entirely changed, there being not the slightest lividity of the face, or frothing of the mouth," &c., &c.

In the next place, I proposed tracheotomy for the paralytic laryngismus which follows the severest attacks of epilepsy instantly endangering life; and by it life has been preserved already in many cases, beginning with that of Mr. Cane, and for the present, ending with that of Dr. Williams, of Wrexham. I say this, notwithstanding the fact that the latter patient died afterwards; for he was rescued from a first danger to succumb to a second and totally different one.

To render the proof of the efficacy of tracheotomy still more complete, it has been observed that the fits have been slighter or more severe, according as the tracheal tube was freely patent, or obstructed by mucus—an event, from its tenacity, very apt to occur. In one case the tracheal tube fell out, the fit was severe, and the patient expired.

The effect of tracheotomy in spasmodic laryngismus is only discovered in the induced absence of fits, or in the mitigated form of the fits which do occur. In paralytic laryngismus it is different; the lividity of the countenance, the distension of the veins, the dyspnœa subside a vue d'æil: the effect is immediate, and beyond a doubt. The danger averted; life preserved.

In this case all depends on the early institution of the operation. The laryngismus arises from paralysis of the laryngeal nerves. But other branches of the pneumogastric are paralysed, and if this condition has long subsisted, the bronchial tubes become clogged with mucus, and the patient dies of bronchial, after having escaped from laryngeal, asphyxia. This was the event in Dr. Herrick's case. It occurs, too, undetected, in many cases in which the laryngismus has subsided, and for whom a vain hope is therefore entertained. I visited such a patient many years ago with my friend Dr. Webster; the fit had passed away, but a diffused bronchial rattle remained, and the patient died of bronchial asphyxia.

I have much to say hereafter on the subject of irritation and of paralysis of the pneumogastric nerve in all its branches, events still unrecognised by the profession. But I now resume the topic of this paper.

Amongst the questions in regard to epilepsy, two most important ones have never yet been considered; the first what are the precise forms of the epilepsia trachelea, and what are the abortive forms of epiplepsy when the effects of laryngismus have been superseded by tracheotomy in the epilepsia laryngea?—the second, what are the causes and modes of death in epilepsy?

I should conclude that there is usually little danger for mind, limb, or life, in the epilepsia trachelea, judging from the effects of tracheotomy in mitigating this danger in the cure of epilepsia laryngea. In several cases not only have the complexion and the general appearance and the general health been greatly improved by tracheotomy, but the impaired mind itself has become restored. This was remarkably the case in Mr. Mackarsie's, Dr. Neill's, and Dr. Bucknill's patients.

The sources of danger to life in epilepsy are—apoplectic asphyxia and, as in tetanus, exhaustion of the nervous powers—that is, coma, and laryngeal or bronchial asphyxia from paralysis of the pneumogastric nerves, and exhaustion and sinking from the violence and repetition of the attacks.

All these are questions, not for hasty and superficial criticism, but for patient and careful investigation. And now to the proper subject of this communication.

There are TWO cases of epilepsy in which the propriety and efficacy of tracheotomy admit of no *doubt*.

The *first* of these is the *epilepsia laryngea*, of inorganic origin, in its early stage, threatening mind or life, not yet involving organic change; that is, *spasmodic* laryngisums aud its effects.

The same remark applies to other convulsive diseases, perpetual convulsion inclusive.

The *second* is, the epilepsia laryngea, proceeding to coma and paralytic laryngisums or stertor, augmenting the coma and endangering life.

The same remark applies to to the coma left by other convulsive diseases, and to all kinds of simple apoplexy; that is, apoplexy without lesion or rupture of vessels, deep intoxication, narcotic poisoning, &c.

It may now be well to take a brief retrospect, and consider what has been done.

Of Mr. Cane's case, the first and most brilliant of all, life was immediately saved, and the other patient was preserved afterwards —notwithstanding some indiscretions, such as occasionally closing the trachea—from his attacks, which had previously occurred on an average five times in a fortnight.

The case of Mr. Anderson was one of the greatest inveteracy, and tracheotomy was performed altogether without diagnosis, for epilepsy not for laryngisums. Yet was its violence mitigated.

In Dr. Neill's case there was great improvement. On one occasion "he was threatened with an attack, of which he was conscious;" "removed a temporary plug," and "the symptoms disappeared!" "He made arrangements to renew his business; walked about the streets in the confidence and consciousness of a strength of mind and purpose which he had not experienced for a long period."

On another occasion a seizure occurred; the treacheal tube, it is supposed, dropped out, and the patient "died almost instantaneously."

In Dr. Herrick's case, the convulsions, after recurring every hour or two from three o'clock to nine o'clock P.M., "kept recurring every twenty or thirty minutes; the face and lips were swollen and livid; the breathing much obstructed by mucus, slow and stertorous; the veins of the face and neck much distendeded." It was concluded that "the patient could not survive an hour unless relieved." Tracheotomy was performed, and followed by "a most marked improvement," "the breathing being more natural, the countenance more livid." On the two following days there were only four convulsions, and these less protracted and severe than any previous to the operation.

Unfortunately, this patient was only relieved. He died on the third day of bronchial asphyxia.

Henceforth let us carefully select our cases. Let a just diagnosis be instituted; let there be a fair degree of hope; let the disease be laryngeal, of sufficient gravity to justify the remedy, but not inveterate, not yet involving organic changes.

I conclude by repeating that there are two cases of epilepsy in its direst forms, in which the propriety and efficacy of trcheotomy admit of no doubt: These are—first, epilepsia laryngea, with spasmodic laryngismus, threatening the extinction of mind; second, epilepsia laryngea, with paralytic laryngismus, threatening the extinction of life.

I now propose to add notes of the recent events which have illustrated the use of tracheotomy in epilepsy. These are given in the words of the observers themselves, who have pursued the inquiry.

The first document is a note of Mr. Mackarsie:—

The following simple detail will not fail to interest your readers. It affords proof of the value of tracheotomy in epilepsy laryngea, by contrasting the condition of the patient before, during and after the existence of the tracheal opening. Had this orifice been maintained freely patent, it is obvious that the loss of intellect first, and the loss of life next, would, humanly speaking have been averted!

The present note completes the account of one of the most interesting of the cases in which tracheotomy has hitherto been performed in epilepsy; and, as the able and judicious writer justly observes:—"there is much that is important to be learnt from it." There is much to be learnt from the following striking paragraphs:—

Clay-cross, September 20th, 1854.

"My Dear Sir,—For many months I have been anxious to communicate to you the results in my case of epilepsy treated by tracheotomy.

"When I last wrote to you, my patient had a recurrence of fits, but in a much mitigated form; his mind had improved, and his complexion being of a dusky hue had assumed a natural colour, only remaining perhaps a little paler than natural.

"At this period the tracheal opening was allowed to close through neglect, and the fits resumed the same form they had had before. I wished to re-open the orifice into the trachea, but was not permitted to do so. I watched my patient with intense interest, and observed him revert to his former condition; the fits became more severe, and the coma more prolonged, the dusky hue of his complexion returned and his mind again gave way, until at length I found it necesssary to remove him to an asylum.

"In this asylum my patient remained a few days, and eventually died from the frequent recurrence of the fits.

"I think there is much of importance to be learnt from this interesting case."

I shall always consider it a great honor to correspond with you.

"I remain, dear Sir, yours very faithfully,

"W. J. MACKARSIE.

"To Dr. Marshall Hall."

The second document I adduce is an extract from an able note from Dr. Bucknell, full of the deepest interest:

"Devon County Lunatic Asylum, Exminster, Sept. 24th 1854.

"MY DEAR SIR,—Both cases on which I operated for tracheotomy have died: one of phthisic, the other of epilepsy. The mode of death in the latter was very instructive. It was the second case mentioned in my paper in THE LANCET in 1853.—

"* * * * The wonderful change in the character of the fit I have described continued for a period of about nine months.—About that time her sister paid her a visit, and they had a quarrel about their mother's will. The day afterwards she was distinctly hysterical, and iu the following night she had severe epileptic convulsions. These continued at intervals of ten minutes for about sixteen hours, when she died from exhaustion, (failure of the heart's action, I suppose,) distinctly not from coma. During the whole of this period, the tracheal opening was clear.—During each fit, the external muscles of respiration could be felt in a state of tonic spasm; and it is probable that the diaphragm was also fixed. For a short period in each fit the respiration was completely arrested. I kept the patient sitting in an easy chair, to prevent the accumulation of mucus in the air-tubes.

"I quote from memory but have the case ready for publication.

"I remain, my dear Sir, yours very truly,

"J. C. BUCKNELL.

"Dr. Marshall Hall."

I have for some time been investigating the causes of death in convulsion and apoplectic diseases. The case of which a note has been given was evidently one of exhaustion of the vis nervosa. In this manner tetanus seems to destroy life. Each successive fit adds to the exhaustion induced by the previous ones, until the patient expires.

If two frogs be affected with strychnine, and one be excited, and the other left in perfect tranquility, the former dies, and the latter survives.

The different forms assumed by epilepsy require to be investigated—the tracheal, laryngeal, the abortive in cases in which tracheotomy is instituted, the syncopal, and not least, this of nervous exhaustion.

It is difficult to imagine how the respiration can be arrested without closure of the glottis. There are three modes, however, in which this may occur: one is, at a full expiration; the second, at a full inspiration; the third, the muscles of inspiration and the muscles of expiration being simultaneously and equally contracted.

In such cases the diagnosis must be established by observing the state of the larynx, of the neck, of the face, and of the cerebrum. In the absence of the laryngismus, the deep purple lividity and tumefaction, and the subsequent deep coma, &c., are equally absent, and tracheotomy of course hors de propos.

Much observation is still required to complete our knowledge of the forms of epilepsy.

The third document I beg to adduce, is an extract from an able note from Dr. Edwards.

"Grosvenor-street, Cheltenham, Sept. 26th, 1854.

"My Dear Sir,— * * * The effect of the operation was immediate, self-evident, axiomatic, to all present. Dr. Allardyce expressed his opinion then, and repeated it this morning, that not only were the effects such, but 'that without it the case must have been utterly hopeless.' * * *

"I may sum up here, that no fit of any severe character took place in my patient from the moment the tracheal opening was made, so long as the tube, of whatever kind or form, was kept perfectly clear, and so long as it was kept in the trachea; and that whenever a seizure of a severe character occurred, the tube was found invariably more or less obstructed.

"Slight abortive fits occurred; but these were so dwindled into insignificance, that the patient, having occasionally but a momentarily feeling, said he was quite well.

"The tube was now removed by the patient. The patient continued well until December, when he became comatose, sank, and died, the history of the attack being wanting.

"Believe me, dear Sir, very faithfully yours,

"Charles Edwards."

I have taken the liberty of abbreviating the last paragraph of Dr. Edwards' admirable note.

Dr. Edwards has done great good to the cause by a most important discovery, for which I feel deeply indebted to him. This is to be found in detail in *The Lancet* for 1853. It is, that the tracheal tube, and even the exterior tube when an interior one is withdrawn, is extremely apt to be clogged and obstructed by viscid mucus. A fit may then occur, and be as severe as if the operation had never been performed.

The case requires no commentary. Tracheotomy has saved—mind and life! What can be added to such a statement?

I will now beg your readers' attention to a few short paragraphs, embracing the principles which should guide us in the use of this heroic remedy in epilepsy; they are five in number:

First.—The treatment by tracheotomy should, as in all other cases of the use of important remedies, be founded on an accurate diagnosis; the case should be unequivocally laryngeal.

Secondly.—The case should admit of remedy; it should not have have been organic in its origin, or have sustained organic change in its progress.

Thirdly.—Not only should there be no organic diseases, but the case should not be inveterate, even if it still be one of function.

For this reason I do not think the cases already consigned to an asylum or the workhouse can offer much promise of permanent benefit from tracheotomy, or from any mode of treatment. The cases must be of such date as to present hope. They must be so severe and threatening in regard to mind or life, as to present sufficient cause or reason for the operation. It will be readily understood that I do not expect the lunatic asylum or the workhouse often to furnish cases for tracheotomy. The cases in these institutions are generally already inveterate or organic.

Fourthly.—The tracheal aperture should be ample, and be sustained unequivocally FREE.

Fifthly.—Our expectations should be reasonable; we should expect to remedy laryngismus and its effects, whatever these may be, and nothing more.

If these five principles guide us, there will be an end of all discreditable controversy; the remedy will have a fair and unprejudiced trial; we shall rejoice if it prove successful, and mourn if it prove a failure.

For myself I have only to say, that I have not, in any case in which tracheotomy has been performed, had the opportunity of forming the diagnosis for myself, having never had occasion to witness a paroxysm in any of them. I believe no proper diagnosis has been established in some of them—in Mr. Anderson's for example. And what shall I say of Dr. Andrea Verga's case? Is it not lamentable to see such things brought forward on so grave an occasion?

I fear that that will long be said of tracheotomy in epilepsy which is constantly said of other operations—as that for hernia—that when instituted, it is instituted too tardily, too late. Another observation may be made; I know positively that the operation has not generally been efficient, for want of an ampler orifice, freely sustained.

Now, let us imagine a youth attacked by epilepsy—by epilepsial laryngea; let us observe that, after a certain number of these attacks, the mind begins to fail; that there is incoherence of ideas, loss of memory, &c.; that this state of things is augmenting; that the patient is in danger of becoming a maniac or an idiot; that all other remedies have failed; that tracheotomy affords a hope. Who will hesitate to perform so simple an operation?

Or, let us imagine that, after a severe epileptic attack, the patient remains in a state of coma, his life being obviously endangered, as in the case of Mr. Cane, Dr. Herrick, Dr. Williams, &c., who will hesitate to avert this danger by performing tracheotomy? In all these three cases the danger was so averted.

I will only add, that as I understand the question better, my hopes of success become more, not less, sanguine. Only let the remedy be instituted in cases perfectly appropriate, after a just and adequate diagnosis, and in a manner perfectly efficient.

